# Changes in Nutritional Habits and Lifestyles Associated With COVID-19 in Jazan, Saudi Arabia: A 2022 Cross-Sectional Survey

**DOI:** 10.7759/cureus.65425

**Published:** 2024-07-26

**Authors:** Mohammed A Muaddi, Abdullah A Alharbi, Mohamed Salih Mahfouz, Reem T Hadadi, Rehaf A Areeshi, Huda K Muqri, Elaf J Zurayyir, Fatimah S Alkuaybi, Shorog A Alhazmi, Muayad S Albadrani, Rawan K Alharbi, Ahmad Y Alqassim

**Affiliations:** 1 Department of Family and Community Medicine, Faculty of Medicine, Jazan University, Jazan, SAU; 2 Faculty of Medicine, Jazan University, Jazan, SAU; 3 Department of Family and Community Medicine and Medical Education, College of Medicine, Taibah University, Medina, SAU; 4 Department of Family Medicine, Al-Husseini Primary Care Center, Western Sector, Jazan Health Cluster, Ministry of Health, Jazan, SAU

**Keywords:** lifestyle change, public and environmental health, preventive medicine, jazan, covid-19 pandemic, physical exercise, dietary habits

## Abstract

Background: The COVID-19 pandemic impacted dietary habits and physical activity patterns, with some long-term consequences. This study evaluated the effects of the pandemic on adults' dietary habits and physical activity in Jazan and compared them to pre-pandemic.

Methods: An analytical cross-sectional survey was conducted among conveniently selected 559 individuals in Jazan, Saudi Arabia, in February 2022 using a validated online questionnaire. Data was collected to assess changes in eating habits, food intake, and weight before and 21 months after lifting of COVID-19 curfew restrictions in the region. Chi-square and McNemar's tests were used for analysis.

Results: The proportion of individuals consuming homemade meals decreased from 50.6% (n=283) before the pandemic to 46.5% (n=260) during the pandemic, while the proportion of participants consuming less than three meals per day increased from 42.2% (n=236) to 45.4% (n=254), and breakfast consumption decreased significantly from 58.1% (n=325) to 53.5% (n=299) (p = 0.033). There was an increase in the consumption of fast food from 10.7% (n=60) to 12.0% (n=67) and dining at restaurants from 18.4% (n=103) to 19.3% (n=108); however, these increases were not statistically significant compared to pre-pandemic rates. During the pre-COVID-19 period, 46.9% (n=262) reported engaging in physical activity one to three times a week, whereas this frequency decreased to 41.3% (n=231) during the pandemic (p = 0.017). In contrast, a significant increase was observed in the duration of computer usage as prior to the pandemic, 20.2% (n=113) reported spending more than five hours per day on the computer, while this proportion increased to 31.8% (n=178) during the pandemic (p < 0.001). Furthermore, a considerable proportion of both males and females, constituting over one-third (n=189) of the total sample, reported an increase in body weight.

Conclusion: The findings suggest that the adult population in the Jazan region of Saudi Arabia experienced significant lifestyle changes during the COVID-19 pandemic, including altered dietary patterns and a significant decline in physical activity. To mitigate potential adverse effects on future well-being, it is crucial to implement enduring initiatives promoting healthy lifestyles.

## Introduction

The COVID-19 pandemic has brought about unprecedented changes to people’s daily lives, including disruptions to dietary habits and physical exercise patterns [1-3]. With lockdowns, social distancing measures, and the closure of many gyms and fitness centers, individuals have been forced to adapt to new ways of living [4-6]. Concurrently, the pandemic's impact on mental well-being cannot be overlooked, as heightened levels of stress and anxiety have become prevalent among individuals navigating these challenging circumstances [6,7].

Stress and anxiety caused by the pandemic can also lead to negative changes in dietary habits and physical exercise patterns [8]. Individuals may turn to comfort eating or neglect their physical health as a way of coping with the added stress and uncertainty [9]. The changes in dietary habits and physical exercise patterns adopted during the pandemic raise a major concern regarding their potential long-term consequences. There is a risk that these changes may become ingrained habits, resulting in weight gain [1,4,10] decreased fitness levels [10,11], and an increased risk of chronic diseases such as heart disease and diabetes [6,12].

Dietary habits and physical exercise are crucial determinants of overall health and well-being [12,13]. Various factors have been identified as influential in shaping these behaviors, including knowledge levels, social factors, and the presence of extended family members in the household [14,15]. Studies have shown that individuals with a higher degree of knowledge regarding healthy eating and physical activity tend to engage in these behaviors more frequently [9,16]. Furthermore, social factors, such as having a supportive network of friends and family members who engage in healthy behaviors, as well as living with extended family members, have been found to exert a substantial positive influence on an individual's dietary habits and physical exercise patterns [14,15].

The existing body of literature has primarily focused on the short-term effects of the COVID-19 pandemic on dietary habits and physical activity patterns, leaving a gap in understanding the long-term impact [4,10]. The changes in behavior that occurred during the pandemic have the potential to exert long-term consequences on health and well-being [3,17], although this potential remains insufficiently explored. Further investigation is necessary to comprehensively understand the long-term impact of the COVID-19 pandemic on dietary habits and physical activity patterns. It is important to recognize that not all effects are negative, as some individuals may have developed healthier habits during the pandemic, such as increased home cooking and engaging in outdoor physical activities [3,5,17-19]. The objective of this study is to evaluate the effects of the COVID-19 pandemic on dietary habits and physical activity patterns among adults in Jazan while comparing these patterns to pre-pandemic norms. This information holds significant importance in the formulation of interventions and strategies aimed at improving dietary habits and enhancing physical activity levels, ultimately contributing to enhanced overall health and well-being.

## Materials and methods

Study design, setting, and participants

This cross-sectional study aimed to investigate the lifestyle changes pertaining to current dietary habits and physical activity resulting from the COVID-19 pandemic. The research was conducted in the Jazan region of Saudi Arabia, situated in the southwestern part of the country along the Red Sea. The capital of the region is Jazan, encompassing 14 governorates and administrative centers. In terms of geographical size, Jazan is the second smallest region among the 13 regions of Saudi Arabia, covering an area of approximately 13,457 square kilometers. As of the Saudi Population Census of 2021, the estimated population of Jazan was approximately 1,355,099 individuals [20].

The target sample frame for this study comprised adults aged 18 years and older residing in the Jazan region. While population statistics are provided for the total Jazan population, the sample frame included only adult residents of this region. The main inclusion criteria for participation in the survey included being a resident of the Jazan region, possessing Arabic language proficiency for effective communication, and expressing willingness to provide informed consent to participate in the study.

The questionnaire provided clear definitions for pre-COVID and post-COVID timeframes. The pre-COVID period was defined as prior to March 2020, before any COVID-19 restrictions or impacts began in the Jazan region. The during-COVID period was defined as current behaviors during the last month from the time of data collection that took place 21 months after lifting of COVID-19 curfew restrictions in the region in February 2022.

Sample size and sample procedure

The sample size for this study was determined using the equation of a cross-sectional survey, where n represents the sample size, p is the anticipated proportion of the phenomena being studied, Z is the standardized variable corresponding to a 95% confidence level, and d is the desired marginal error.

Given the lack of available data on the proportion of adults in the Jazan region who changed their health behaviors during the COVID-19 pandemic, a conservative approach was adopted, assuming a prevalence of 50%. By substituting the values p = 0.5, d = 0.04, and Z = 1.96 into the equation, the initial estimated sample size (n) was calculated as 600.

To account for the potential non-response associated with online surveys, the initial sample size was increased by 10% to 660 adults. Convenient sampling was used to recruit participants and achieve a broad distribution. Participants were invited to complete an anonymous electronic questionnaire created with Google Forms. The questionnaire was distributed for self-administration through WhatsApp, a widely used social media platform in Saudi Arabia.

Method of data collection and study tools

In this study, we utilized a previously validated, multi-component, self-administered questionnaire to collect data [21]. The questionnaire was distributed online using Google Forms in February 2022. The distribution of the questionnaire was facilitated through WhatsApp, the most popular social media platform in Saudi Arabia. The survey was distributed through the authors' social networks via WhatsApp by requesting contacts to further share the questionnaire within their own networks. Data were collected from Arabic-speaking participants aged 18 years or older.

The survey contained various aspects, including sociodemographic information (10 questions), sources of health and nutrition information (two questions), dietary habits (nine questions), and physical activity (four questions). Participants were asked to respond to each question twice, providing information about their behavior both before the COVID-19 pandemic and during the month preceding data collection.

The living situation was originally assessed across seven options. For analysis, this variable was dichotomized into two groups: living with a nuclear family/alone or living with an extended family. The nuclear family/alone group included those living alone, with roommates, with a spouse, or with a spouse and children. The extended family group included those living with parents, with parents and siblings, or with parents, siblings, and children.

The questionnaire clearly defined pre-COVID and during COVID-19 time periods for participants. For pre-COVID, participants were asked about behaviors prior to any COVID-19 impacts and restrictions in March 2020. For during COVID, given data collection occurred in February 2022, participants were asked about their current behaviors over the past month to capture lifestyle habits during the pandemic period. Instructions directed participants to think back to their typical pre-pandemic routines and differentiate them from current habits when answering questions. The survey questions focused on recalling frequencies and habitual behaviors rather than quantitative details to facilitate recall.

Assessment of dietary habits

The questionnaire comprised 10 questions aimed at assessing the frequency of consumption of specific food groups during the COVID-19 pandemic. These food groups were categorized into five distinct groups: frozen foods, homemade foods, fast food, healthy foods, and restaurant-prepared meals. These categories included a variety of dietary choices, including traditional Mediterranean food as well as high-sugar and high-fat consumed meals that have recently gained popularity in the kingdoms of Saudi Arabia [3]. The questionnaire targeted the following specific food groups: fruits, vegetables, milk and dairy products, meat and meat products (including red meat, chicken, and fish), grains (such as bread, rice, and pasta), sweets, sugar-sweetened beverages, coffee, tea, and energy drinks.

Assessment of physical activity

The assessment of physical activity patterns among study participants involved the inclusion of four questions, which were adapted from the International Physical Activity Questionnaire Short Form (IPAQ-SF) [22]. Participants were asked on two occasions regarding their exercise frequency per week, the number of times they engaged in household chores per week, the daily duration of computer usage for work or studying, and the daily duration spent on entertainment activities such as television, computers, and social media. Specifically, participants were asked to provide separate responses for two time periods: the period prior to the pandemic and the current time period.

Data quality control

The face and content validity of the questionnaire items were assessed based on feedback from reviewers, focusing on the relevance of the items to the subject matter and the time required to complete the questionnaire. The survey underwent further validation through review by healthcare professionals and academic medical staff. Throughout the data collection process, the study team diligently monitored the progress on a daily basis. For the final version of the questionnaire, a satisfactory level of internal consistency reliability was attained, as evidenced by a Cronbach's Alpha coefficient of 0.732.

Data analysis

Data analysis was conducted using IBM SPSS Statistics for Windows, Version 25 (Released 2017; IBM Corp., Armonk, New York, United States). Frequencies and percentages were employed to summarize categorical variables. The chi-square test was utilized to assess the relationship between categorical study variables. McNemar’s test was employed to examine statistically significant changes in category frequencies from pre- to post-pandemic. Statistical significance was determined with a p-value threshold of less than 0.05.

Ethical consideration

Prior to initiating the research, ethical approval was obtained from the Standing Committee of Scientific Research at Jazan University, with the approval number REC-43/05/090, dated 26/12/2021. The study adhered to the fundamental principles outlined in the Declaration of Helsinki, ensuring the protection of participants' rights and welfare. Participation in the study was entirely voluntary, and anonymity was maintained throughout the data collection process. Participants were fully informed about the study objectives and procedures, and they had the freedom to decline participation or withdraw from the study at any stage without facing any consequences. Informed consent was obtained from all participants involved in the study. Participants were required to click on the "I Agree" button to confirm their agreement to participate in the research. Confidentiality of the collected data was upheld, and data was securely stored and accessed only by authorized researchers. The research team was committed to upholding the highest ethical standards to safeguard the rights and well-being of the study participants. The duration of the online survey was between 03/February/2022 and 25/March/2022.

## Results

The respondent sample consisted predominantly of young adult females aged 18-25 years (62.3% and 46.9%, respectively), most of whom had a bachelor's degree (65.1%). Approximately half were married (48.3%), while a similar proportion were single (47.9%). The majority lived with a spouse and children (39.2%). More than half (54.9%) did not work or study remotely during the pandemic. Regarding children studying from home, over one-third had none (35.4%), while 27.9% had 3 or more. Overall, one-third reported weight gain (33.8%) and 31.1% described their health as very good amidst the pandemic restrictions (Table 1).

**Table 1:** Demographic characteristics of study participants (n =559).

Characteristics		Frequency	Percentage (%)
Gender	Male	211	37.7
	Female	348	62.3
Age groups (years)	18-25	262	46.9
	>25-35	109	19.5
	>35-45	132	23.6
	>45-55	49	8.8
	>55	6	1.1
Marital status	Married	270	48.3
	Single	268	47.9
	Divorced	19	3.4
	Widowed	2	0.4
Number of children studying from home	None	198	35.4
	1	109	19.5
	2	96	17.2
	≥ 3	156	27.9
Education level	Less than high school	11	2.0
	High school	122	21.8
	Diploma	47	8.4
	Bachelor`s degree	364	65.1
	Higher than bachelor`s degree	15	2.7
Employment status	Full-time	212	37.9
	Part-time	10	1.8
	Unemployed	81	14.5
	Retired	14	2.5
	Self-employed	6	1.1
	Student	236	42.2
Working/studying from home	Yes	209	37.4
	No	307	54.9
	Not applicable	43	7.7
Living arrangements	Alone	14	2.5
	With roommates	7	1.3
	With spouse (husband or wife)	32	5.7
	With spouse and children	219	39.2
	With parents	22	3.9
	With parents and siblings	210	37.6
	With parents, siblings, and children	55	9.8
Weight change during the pandemic	Gained weight	189	33.8
	Lost weight	96	17.2
	Maintained weight	222	39.7
	Do not know	52	9.3
Perceived health status during the last two months	Excellent	160	28.6
	Very good	174	31.1
	Good	98	17.5
	Fair	100	17.9
	Poor	27	4.8

**Figure 1:**
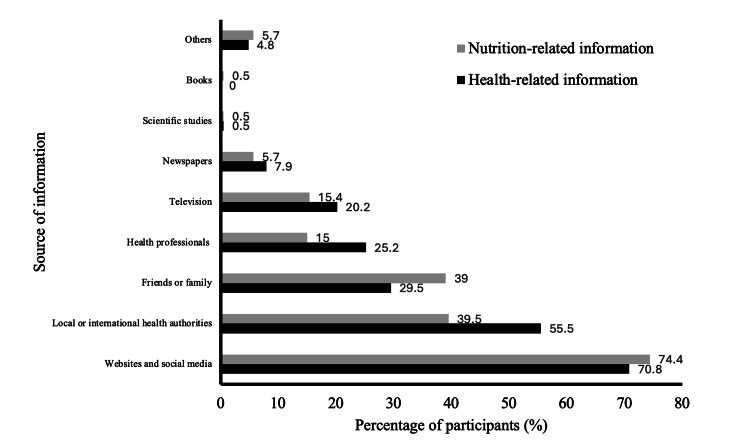
Source of health and nutrition-related information during the COVID-19 pandemic (n =559). Participants were able to select multiple sources of health and nutrition information.

The most common source of both health and nutrition information was websites and social media, reported by 70.8% and 74.4% of participants, respectively. Local and international health authorities were also used by a considerable proportion of respondents for health and nutrition information (55.5% and 39.5%, respectively). On the other hand, books and scientific articles were the least commonly utilized sources (Figure 1).

Homemade meal consumption decreased by 4.1% during the pandemic compared to pre-pandemic. Fast food intake increased by 1.3% and restaurant dining increased by 0.9%, though not significantly. Consuming <3 meals/day rose 3.2%, while 3 meals/day dropped 4.5%. Breakfast consumption decreased significantly by 4.6% (p=0.033), with breakfast skipping increasing 4.6%. Daily water intake patterns changed, with 5-7 cup and >8 cup consumption increasing by 2.1% and 3.0%, respectively (Table 2).

**Table 2:** Eating habits before and during the COVID-19 pandemic (n =559). n: Sample size; McNemar's test was used to evaluate statistically significant changes in category frequencies from before to during the pandemic.

Nutrition	Variables	Pre-COVID-19 n (%)	During COVID-19 n (%)	p-value
Food consumption	Frozen ready to eat	16 (2.9)	24 (4.3)	0.152
	Fast food	60 (10.7)	67(12.0)	0.483
	Restaurants	103 (18.4)	108 (19.3)	0.714
	Healthy food	97 (17.4)	100 (17.9)	0.801
	Home cooked	283 (50.6)	260 (46.5)	0.054
Number of meals	Less than three meals	236 (42.2)	254 (45.4)	0.130
	Three meals	243 (43.5)	218 (39.0)	0.050
	More than three meals	80 (14.3)	87 (15.6)	0.520
Eating breakfast	Yes	325 (58.1)	299 (53.5)	0.033
	No	234 (41.9)	260 (46.5)	
Skipping meals	Yes	438 (78.4)	433 (77.5)	0.712
	No	121 (21.6)	126 (22.5)	
Number of water glasses	1-4 glasses	300 (53.7)	271 (48.5)	0.008
	5-7 glasses	167 (29.9)	179 (32.0)	0.327
	≥ 8 glasses	92 (16.5)	109 (19.5)	0.040

Overall, the majority of participants consumed most food groups just once daily. However, intake of tea/coffee and bread/rice/noodles was higher, with 45.3% and 35.6% consuming these more than two times daily, respectively. Fruit and vegetable consumption was low, with only 60.3% and 60.1% eating these once daily, and many never consuming them (31.8% and 17.9%, respectively). Milk and dairy followed this trend. Animal protein intake was slightly better at 64.9% once daily. Energy drinks were the least consumed item, with 70.5% never drinking them (Table 3).

**Table 3:** Self-reported frequency of consumption of selected types of food during the COVID-19 pandemic (n =559).

Food items	> 3 Times per day n (%)	2-3 Times per day n (%)	Once per day n (%)	Never
Fruits	8 (1.4)	36 (6.4)	337 (60.3)	178 (31.8)
Vegetable	11 (2.0)	112 (20.0)	336 (60.1)	100 (17.9)
Milk and dairy products	23 (4.1)	96 (17.2)	338 (60.5)	102 (18.2)
Meat/chicken/fish	22 (3.9)	136 (24.3)	363 (64.9)	38 (6.8)
Bread/rice/noodle	32 (5.7)	167 (29.9)	329 (58.9)	31 (5.5)
Tea or coffee	78 (14.0)	174 (31.3)	239 (42.8)	68 (12.2)
Sweet drinks	36 (4.6)	106 (19.0)	283 (50.6)	134 (24.0)
Energy drinks	14 (2.5)	25 (4.5)	126 (22.5)	394 (70.5)

**Figure 2:**
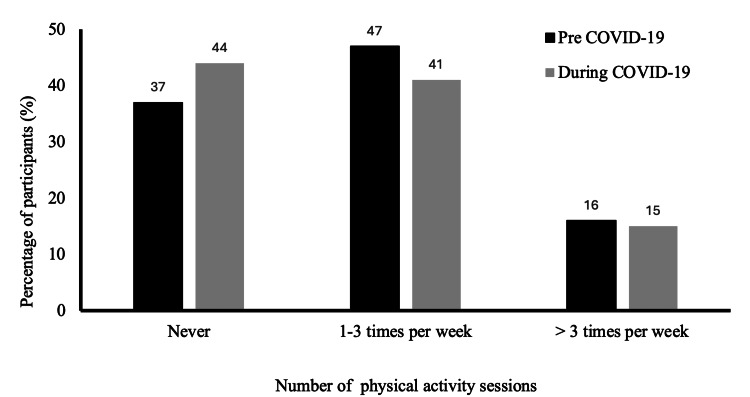
Self-reported physical activity before and during the COVID-19 pandemic.

Physical activity engagement declined during the pandemic, with the proportion of exercising 1-3 times weekly dropping from 46.9% pre-pandemic to 41.3% during COVID-19 (p=0.017) (Figure 2). Time spent on sedentary behaviors increased significantly, including more than 5 hours daily on the computer rising from 20.2% to 31.8% (p=0.001) and more than 5 hours on TV/social media increasing from 32.7% to 39.5% (p=0.001). Engagement in household chores 1-3 times weekly, 4-5 times weekly, or daily did not change significantly between the pre-pandemic and pandemic periods, with around two-thirds of participants reporting chore engagement within these frequency ranges during both timeframes (Table 4).

**Table 4:** Self-reported daily activities pre- and during the COVID-19 pandemic (n =559). n: Sample size; McNemar's test was used to evaluate statistically significant changes in category frequencies from before to during the pandemic.

Factors	Pre-COVID-19 n (%)	During COVID-19 n (%)	p-value
Doing exercise			
Never	207 (37.0)	244 (43.6)	0.004
1-3 times weekly	262 (46.9)	231 (41.3)	0.017
More than 3 times weekly	90 (16.1)	84 (15.0)	0.515
Doing household chores			
Never	185 (33.1)	168 (30.1)	0.027
1-3 times/week	189 (33.8)	205 (36.7)	0.078
4-5 times/week	35 (6.3)	42 (7.5)	0.243
Daily	150 (26.8)	144 (25.8)	0.337
Time spent on a computer			
None	166 (29.7)	119 (21.3)	<0.001
1-2 hours	155 (27.7)	117 (20.9)	<0.001
3-5 hours	125 (22.4)	145 (25.9)	0.089
More than five hours	113 (20.2)	178 (31.8)	<0.001
Time spent on television, computers, social media for entertainment			
Less than 30 minutes	59 (10.6)	51 (9.1)	0.157
1-2 hours	156 (27.9)	119 (21.3)	<0.001
3-5 hours	161 (28.8)	168 (30.1)	0.518
More than five hours	183 (32.7)	221 (39.5)	<0.001

Meals per day did not change significantly before versus during the pandemic across gender, age, living arrangements, or children studying from home (Table 5). However, significant changes were seen in weight and physical activity. A greater proportion of females (23.1%) reported weight loss compared to males (12.5%) (p=0.006), alongside a greater proportion of young adults aged 18-35 years (22.1%) versus those over 35 years (13.0%) (p=0.043). Females (24.1%) also reported reduced physical activity compared to males (18.0%), though not significantly. Young adults aged 18-35 years (24.7%) reported decreased activity versus those over 35 years (16.0%) significantly (p<0.001).

**Table 5:** Lifestyle changes during the COVID-19 pandemic according to selected characteristics (n=559). Living arrangements: Dichotomized as living with nuclear family/alone (alone, with roommates, with spouse, with spouse and children) or living with extended family (with parents, with parents and siblings, with parents, siblings, and children); n: Sample size; p-values were obtained by the chi-square test

			Pattern of change		
			Number of Meals		
Characteristics		Decreased n (%)	Unchanged n (%)	Increased n (%)	p-value
Gender	Male	30 (14.2)	155 (73.5)	26 (12.3)	0.061
	Female	67 (19.3)	222 (63.8)	59 (17.0)	
Age	18-35 years	69 (18.5)	240 (64.5)	63 (16.9)	0.105
	More than 35 years	28 (15.0)	137 (73.3)	22 (11.8)	
Living arrangements	With nuclear family/ Alone	46 (16.0)	203 (70.7)	38 (13.2)	0.219
	With extended family	51 (18.8)	174 (64.0)	47 (17.3)	
Number of studying children from home	No Child	40 (20.2)	129 (65.2)	29 (14.6)	0.420
	One or more	57 (15.8)	248 (68.7)	56 (15.5)	
			Weight		
		Decreased n (%)	Unchanged n (%)	Increased n (%)	p-value
Gender	Male	25 (12.5)	100 (50.0)	75 (37.5)	0.006
	Female	71 (23.1)	122 (39.7)	114 (37.1)	
Age	18-35 years	73 (22.1)	138 (41.8)	119 (36.1)	0.043
	More than 35 years	23 (13.0)	84 (47.5)	70 (39.5)	
Living arrangements	With nuclear family/ Alone	36 (14.0)	122 (47.5)	99 (38.5)	0.014
	With extended family	60 (24.0)	100 (40.0)	90 (36.0)	
Number of studying children from home	No Child	29 (16.6)	79 (45.1)	67 (38.3)	0.615
	One or more	67 (20.2)	143 (43.1)	122 (36.7)	
			Physical Activity		
		Decreased n (%)	Unchanged n (%)	Increased n (%)	p-value
Gender	Male	38 (18.0)	135 (64.0)	38 (18.0)	0.118
	Female	84 (24.1)	213 (61.2)	51 (14.7)	
Age	18-35 years	92 (24.7)	211 (56.7)	69 (18.5)	<0.001
	More than 35 years	30 (16.0)	137 (73.3)	20 (10.7)	
Living arrangements	With nuclear family/alone	55 (19.2)	194 (67.6)	38 (13.2)	0.026
	With extended family	67 (24.6)	154 (56.6)	51 (18.8)	
Number of studying children from home	No Child	54 (27.3)	112 (56.6)	32 (16.2)	0.056
	One or more	68 (18.8)	236 (65.4)	57 (15.8)	

## Discussion

The COVID-19 pandemic has had a significant impact on the daily lives of individuals worldwide, altering their dietary habits and physical activity patterns. However, the extent to which this trend has persisted beyond the pandemic remains uncertain. This study investigated the long-term influence of the pandemic on the dietary habits and physical activity of adults residing in the Jazan region of Saudi Arabia, 21 months following the lifting of curfew restrictions in May 2020 [23]. The findings revealed that more than one-third of the participants experienced weight gain during the study period. Additionally, social media platforms and websites emerged as the primary sources of health and nutrition information for the participants, whereas books and scientific studies were the least utilized sources. Interestingly, a significant increase was observed in both the proportion of participants skipping breakfast and the proportion of participants with increased water consumption. In terms of physical activity, a significant overall decrease was noted, except for individuals who used to regularly engage in physical activity, as their levels remained unchanged. Moreover, participants reported a significant increase in computer usage for both work-related tasks and leisure activities.

Our study found that books and scientific articles are rarely utilized as sources of information [24]. Over 70% of participants primarily rely on websites and social media for health and nutrition information, while a substantial proportion (55.5% for health; 39.5% for nutrition) also turn to local or international health authorities (Figure 1). This suggests that individuals seek accurate information from official sources during crises. Considering the rapid dissemination of misinformation and rumors in the era of social media, which has emerged as the dominant information source [25], relying on credible sources becomes crucial for accurate and reliable guidance during a pandemic [26]. The significant proportion of individuals using trustworthy sources, such as health authorities, is reassuring. Although social media can be unreliable, it also serves as an effective tool for scientific communication, promoting evidence-based information and healthy behaviors, particularly among younger generations who heavily rely on these platforms during increased screen time.

Our study participants reported no change in the overall number of meals consumed per day. However, they did report an increase in the prevalence of skipping breakfast (Table 2). In contrast, findings from other studies conducted in the UAE [7] and Turkey [27], reported an increase in the number of meals consumed per day and a decrease in the percentage of individuals skipping meals, particularly breakfast, during the pandemic. However, another study conducted in Turkey after lifting the curfew [12] found an increase in skipping breakfast that was attributed to changes in sleep patterns and late waking times. Conversely, during the lockdown in Turkey [27], participants had more regular breakfast, which they previously skipped due to pre-pandemic work status and time constraints. Contradictions between studies on meal consumption patterns during the COVID-19 pandemic may arise from cultural and socioeconomic differences, diverse demographics influencing dietary habits and meal-skipping tendencies, and variations in the timing of studies in relation to public health measures. These factors might impact meal consumption patterns differently across countries.

A 2020 study conducted in Saudi Arabia reported a significant increase in home-cooked food consumption, which was attributed to factors such as restaurant closures, concerns about contracting the virus, and economic uncertainties prompting cost-saving measures [2]. The data shows that the proportion of participants consuming home-cooked meals was lower during the pandemic compared to before the pandemic. This indicates there was a decrease in home-cooked meal consumption during COVID-19 compared to pre-pandemic levels. This finding is consistent with other research exploring the effects of the pandemic on dietary habits during lockdowns. For example, a nationwide survey carried out by researchers at King Saud University documented a relative decrease in home-cooked food consumption [28]. Although home cooking remained the most commonly reported source of food during this period, the proportion decreased compared to pre-pandemic levels. These findings suggest that while the initial phase of the pandemic resulted in an increase in home cooking, this trend may not be sustained in the long term, as individuals begin to revert to their pre-pandemic food habits with the gradual easing of restrictions. Factors contributing to this shift may include the resumption of social activities and dining out, as well as the increased availability of takeout and delivery services facilitated by online platforms and delivery applications. Overall, these findings highlight the intricate interplay between social, economic, and personal factors that influence food habits during a pandemic [29,30]. While the temporary surge in home cooking during lockdown periods can be considered a positive shift, our data suggest that it may not be a sustained trend.

Our study findings indicate that a considerable proportion of both males and females, constituting over one-third of the total sample, experienced an increase in body weight (Table 5). This observation aligns with numerous international studies [31,32] that have also reported similar trends. This prevalence of weight gain has raised global concerns due to its association with an increased risk of overweight, obesity, and related health complications [33]. A recent meta-analysis and systematic review further supported these findings [34], emphasizing the need for comprehensive strategies to address this issue.

We observed a significant increase (17.8%) in the proportion of individuals who reported never engaging in exercise (Table 5), which suggests a marked decline in physical activity levels among a substantial portion of the population. This trend aligns with findings documented in the literature [35-38]. However, participants already committed to regular physical activity demonstrated resilience against the pandemic's disruption to exercise routines, highlighting the importance of maintaining consistent physical activity patterns. Interestingly, a positive shift in activity levels was identified among previously sedentary individuals, with a 14.6% increase in those transitioning to moderate activity following the pandemic. This change may be attributed to heightened awareness of the health risks associated with sedentary behavior during the pandemic.

The impact of COVID-19 on physical activity levels and its relationship to the disease remains incompletely understood. Active individuals may have been more motivated and resilient in adapting to new circumstances, such as finding alternative exercise methods or utilizing online resources. These individuals may have also had more resources available to support their physical activity, such as home exercise equipment or access to virtual fitness programs [35]. Conversely, the pandemic may have presented an opportunity for previously sedentary individuals to adopt a more active lifestyle, driven by changes in daily routines and increased awareness of the importance of physical exercise for health.

Investigating these factors and their implications for promoting physical activity during and beyond the pandemic is crucial. This includes examining the role of intrinsic motivation, resource availability, and the influence of disrupted routines on individuals' decisions to engage in physical activity for their physical and mental well-being. Overall, these findings emphasize the diverse impacts of the pandemic on physical activity levels and underscore the necessity for tailored interventions to encourage and support exercise habits across various population segments.

Several limitations should be acknowledged in relation to our research. Firstly, the use of an online self-administered questionnaire introduces the possibility of response bias, as participants may provide inaccurate or incomplete information. Secondly, our study design was cross-sectional, which allows us to observe associations but does not establish causal relationships between variables. Therefore, caution should be exercised when interpreting the findings. Thirdly, recall bias may have affected participants' abilities to accurately report pre-pandemic behaviors compared to more recent behaviors. Fourthly, it is important to note that our study was conducted in a specific governorate of Saudi Arabia, which may limit the generalizability of the results to other regions or populations. It is essential to consider the context and characteristics of the study population when applying these findings to broader settings. Despite these limitations, our research provides valuable insights into the impact of the COVID-19 pandemic on dietary habits and physical activity. The findings can inform policymakers and healthcare administrators in developing targeted intervention programs that address the specific needs and challenges identified in our study population.

## Conclusions

This research revealed significant lifestyle changes among adults in Saudi Arabia during the COVID-19 pandemic, including increased fast food consumption, meal skipping, decreased physical activity, and increased sedentary behavior. These findings emphasize the need for sustained public health efforts to encourage healthy eating and regular exercise. Interventions should promote nutritious food choices, daily physical activity, and reducing sedentary time through campaigns, guidance on home exercise, access to outdoor spaces, and online fitness resources.

The insights gained are valuable for developing strategies to minimize adverse lifestyle effects of the pandemic. Policymakers, healthcare professionals, and researchers must collaborate to implement effective interventions that improve dietary habits and physical activity levels, promoting long-term health and well-being.
